# TrkB-mediated sustained neuroprotection is sex-specific and $\text{ER}\alpha$-dependent in adult mice following neonatal hypoxia ischemia

**DOI:** 10.1186/s13293-023-00573-0

**Published:** 2024-01-04

**Authors:** Vishal Chanana, Margaret Hackett, Nazli Deveci, Nur Aycan, Burak Ozaydin, Nur Sena Cagatay, Damla Hanalioglu, Douglas B. Kintner, Karson Corcoran, Sefer Yapici, Furkan Camci, Jens Eickhoff, Karyn M. Frick, Peter Ferrazzano, Jon E. Levine, Pelin Cengiz

**Affiliations:** 1 Department of Pediatrics, University of Wisconsin-Madison, 1500 Highland Ave-T503, Madison, WI 53705-9345 USA; 2 Waisman Center, University of Wisconsin-Madison, Madison, WI USA; 3 Department of Biostatistics & Medical Informatics, University of Wisconsin-Madison, Madison, WI USA; 4 Department of Psychology, University of Wisconsin-Milwaukee, Milwaukee, WI USA; 5 Department of Neuroscience, University of Wisconsin-Madison, Madison, WI USA

**Keywords:** Sex differences, Hypoxic ischemic encephalopathy, Hypoxia, Ischemia, Neonate, Brain injury, Estrogen receptor, Tyrosine kinase B, TrkB, 7,8-Dihydroxyflavone, DHF

## Abstract

**Background:**

Neonatal hypoxia ischemia (HI) related brain injury is one of the major causes of life-long neurological morbidities that result in learning and memory impairments. Evidence suggests that male neonates are more susceptible to the detrimental effects of HI, yet the mechanisms mediating these sex-specific responses to neural injury in neonates remain poorly understood. We previously tested the effects of treatment with a small molecule agonist of the tyrosine kinase B receptor (TrkB), 7,8-dihydroxyflavone (DHF) following neonatal HI and determined that females, but not males exhibit increased phosphorylation of TrkB and reduced apoptosis in their hippocampi. Moreover, these female-specific effects of the TrkB agonist were found to be dependent upon the expression of $\text{ER}\alpha$. These findings demonstrated that TrkB activation in the presence of $\text{ER}\alpha$ comprises one pathway by which neuroprotection may be conferred in a female-specific manner. The goal of this study was to determine the role of $\text{ER}\alpha$-dependent TrkB-mediated neuroprotection in memory and anxiety in young adult mice exposed to HI during the neonatal period.

**Methods:**

In this study, we used a unilateral hypoxic ischemic (HI) mouse model. $\text{ER}\alpha$^+/+^ or $\text{ER}\alpha$^−/−^ mice were subjected to HI on postnatal day (P) 9 and mice were treated with either vehicle control or the TrkB agonist, DHF, for 7 days following HI. When mice reached young adulthood, we used the novel object recognition, novel object location and open field tests to assess long-term memory and anxiety-like behavior. The brains were then assessed for tissue damage using immunohistochemistry.

**Results:**

Neonatal DHF treatment prevented HI-induced decrements in recognition and location memory in adulthood in females, but not in males. This protective effect was absent in female mice lacking $\text{ER}\alpha$. The female-specific improved recognition and location memory outcomes in adulthood conferred by DHF therapy after neonatal HI tended to be or were $\text{ER}\alpha$-dependent, respectively. Interestingly, DHF triggered anxiety-like behavior in both sexes only in the mice that lacked $\text{ER}\alpha$. When we assessed the severity of injury, we found that DHF therapy did not decrease the percent tissue loss in proportion to functional recovery. We additionally observed that the presence of $\text{ER}\alpha$ significantly reduced overall HI-associated mortality in both sexes.

**Conclusions:**

These observations provide evidence for a therapeutic role for DHF in which TrkB-mediated sustained recovery of recognition and location memories in females are $\text{ER}\alpha$-associated and dependent, respectively. However, the beneficial effects of DHF therapy did not include reduction of gross tissue loss but may be derived from the enhanced functioning of residual tissues in a cell-specific manner.

## Plain language summary

Periods of low oxygen delivery and blood flow to the brains of newborns are known to cause life-long impairments to their cognitive ability as adults. Interestingly, male newborns are more susceptible to this injury than females. The mechanisms causing this sex difference are poorly understood. Here we test the role of the nerve growth factor receptor tyrosine kinase B (TrkB) in providing long-term neuroprotection following neonatal hypoxia–ischemia (HI) in mice. We have previously shown that when mice are treated with the TrkB agonist 7,8-dihydroxyflavone (DHF) in the days following neonatal HI, the result is short-term neuroprotection only in females and this protection is dependent on the presence of the estrogen receptor alpha receptor ($\text{ER}\alpha$). In this study, we extend these observations by subjecting mice either with or without $\text{ER}\alpha$ to HI. Some of the mice were then treated with DHF immediately after HI. As adults, we performed tests to assess the mice’s memory and anxiety-like behavior. At the end of these tests, we assessed the brains for tissue loss. Our results show that as adults the DHF treatment following HI in neonatal mice preserved memory only in females and this effect was dependent on the presence of $\text{ER}\alpha$. In addition, DHF therapy triggered anxiety-like behavior in mice lacking $\text{ER}\alpha$. We also show that this neuroprotection is not dependent on preservation of brain tissue following the injury. These results provide insight into the mechanisms behind the female resistance to hypoxic ischemic episodes as newborns.

## Highlights

- Female neonates are more resistant to the long-term effects of hypoxic ischemic encephalopathy. The mechanism behind female-specific neuroprotection is poorly understood.
- The preservation of location memory in adult mice subjected to HI as neonates and treated with DHF is specific to females and requires $\text{ER}\alpha$.
- DHF therapy in mice lacking $\text{ER}\alpha$ predisposes both male and female mice to increased anxiety-like behavior.
- This neuroprotection in females does not correlate with the extent of brain tissue damage.

The preservation of location memory in adult mice subjected to HI as neonates and treated with DHF is specific to females and requires $\text{ER}\alpha$.

DHF therapy in mice lacking $\text{ER}\alpha$ predisposes both male and female mice to increased anxiety-like behavior.

## Background

Neonatal hypoxia ischemia (HI) related brain injury is one of the major contributors to postnatal morbidity and mortality [1, 2]. Central nervous system injury caused by oxygen deprivation and decreased blood flow to the neonatal brain around the time of birth can result in devastating life-long mental and physical disabilities [3]. The incidence of neonatal HI in humans ranges from 1 to 8/1000 in developed countries to 26/1000 in developing countries, making HI-related brain injury a global health problem [1]. Recent clinical trials showed that therapeutic hypothermia as a neuroprotective strategy only reduces the risk of death and disability by 11% and leaves 40% of infants with neurological deficits [4]. In fact, the mortality or disability rate at 18–24 months of age in infants with a diagnosis of HI and treated with therapeutic hypothermia is 54% [5]. In addition to therapeutic hypothermia, erythropoietin has been tested in human neonates and found not to lower the risk of death or neurodevelopmental impairments [6]. Thus, understanding the underlying mechanisms of brain-based developmental disorders following HI remains vitally important as means to open new avenues toward the development of new neuroprotective sex-targeted therapies.

The incidence and mortality of HI are two times higher in male neonates compared to females, implicating sex as a risk factor in the severity of HI-induced neural impairment [7]. In addition, male neonates are at greater risk for developing neurological disorders such as autism spectrum disorder, attention deficit disorder, cerebral palsy, blindness, and deafness [8–11]. The underlying mechanisms of sex differences resulting from acute injury leading to neurodevelopmental disorders in neonates with a diagnosis of HI are not fully understood. Experimental studies employing Vannucci's rodent model of HI have suggested that sex differences in the consequences of these injuries may be explained by sex-specific hormones, the presence of X-linked inhibitor of apoptosis protein in females and/or sex-dependent differences in inflammatory responses [12–17].

We previously reported that the TrkB agonist/modulator, 7–8 dihydroxyflavone (DHF), when given 10 min (min) following neonatal HI and then daily for 3 days, increased TrkB phosphorylation and decreased FluroJade-C staining in CA1 neurons only in female hippocampi 3 days post-HI [13]. This acute neuroprotection seen at 3 days in females translates into improved hippocampal-dependent learning and memory in young adulthood (P60 +) assessed by the Morris Water Maze test [13]. Following our initial findings, we conducted experiments in $\text{ER}\alpha$ null mutant mice and discovered that this TrkB-mediated early hippocampal neuroprotection when assessed by cleaved caspase 3 is $\text{ER}\alpha$-dependent in mice 3 days post-HI [18] Thus, the female bias in early TrkB phosphorylation leading to decreased apoptosis post-HI was eliminated in $\text{ER}\alpha$ null mutant mice. However, the role of $\text{ER}\alpha$ in TrkB-mediated neurological recovery from HI is unknown [18]. Thus, the goal of this study was to determine the role of $\text{ER}\alpha$-dependent TrkB-mediated learning and memory as well as anxiety-like behavior as a function of sustained neurodevelopmental outcome in young adult mice exposed to HI during the neonatal period.

## Materials and methods

### Materials

Mouse microtubule associated protein 2 (MAP2) antibody, goat serum, and 7,8-dihydroxyflavone (DHF) were obtained from Sigma (St. Louis, MO). Vectashield mounting media with DAPI was purchased from Vector Laboratories (Burlingame, CA). Goat anti-mouse Alexa Fluor 488-conjugated IgG was obtained from Life Technologies (Carlsbad, CA).

### Animal use

All procedures on mice were carried out in adherence with NIH Guide for the Care and Use of Laboratory Animals using protocols reviewed by the Institutional Animal Care and Use Committee at University of Wisconsin-Madison. After weaning the pups, ear tags were applied (Stoelting, Ear Tag®, Illinois) to track the identification of the individual pup. Then pups were housed 2–4 per cage with food and water provided ad libitum [19]. All animals are maintained in tightly controlled temperature (23 ± 5 °C), humidity (40–50%), and light/dark (12/12 h) cycle conditions in a 200 lx light environment. Prior to behavioral testing, a Mouse Igloo® (Bio-Serv Corporation, Flemington, NJ) was placed to each home cage for general enrichment.

### Genotyping

$\text{ER}\alpha$ heterogeneous ($\text{ER}\alpha$^+/-^) C57BL/6J mice ordered from the Jackson Laboratory were bred at the age of 2 months to obtain complete $\text{ER}\alpha$ knockout mice ($\text{ER}\alpha$^−/−^). Pups were genotyped within 9 days of birth. Genotypes were determined by PCR of genomic DNA from finger or toe clippings. Clippings were heated at 95 °C for 45 min in 50 mM NaOH and neutralized with equal volume of 1 M Tris, pH 6.8. One µL of this DNA solution was added to 19 µL of the following: 0.25 mM of primers for the $\text{ER}\alpha$ gene, 1X GoTaq Buffer (Promega, Madison, WI), 0.2 mM each deoxynucleotide (Promega) and 8 U Platinum Taq (Life Technologies). PCR was performed for 30 cycles as follows: 95 °C for 3 min, denaturation at 95 °C for 30 s, annealing at 58 °C for 30 s ($\text{ER}\alpha$^−/−^ PCR1) or 51 °C for 30 s ($\text{ER}\alpha$^−/−^ PCR2), and elongation at 72 °C for 1 min. PCR products were separated electrophoretically on an ethidium bromide-containing 2% agarose gel and visualized under UV illumination [18].

### Induction of neonatal HI

HI was induced using a model of unilateral hypoxic ischemic reperfusion injury as described previously with some modifications [20]. This is a well-characterized model of neonatal HI and results in reproducible brain injury ipsilateral (IL) to the electrocauterized left common carotid artery [18, 21]. In this model, unilateral sectioning of the common carotid artery alone does not induce ischemic injury due to collateral circulation from the contralateral (CL) side through the circle of Willis. Only subsequent exposure to hypoxia results in hemispheric ischemia as a result of the preferential decrease of blood flow to the IL hemisphere secondary to hyperventilation resulting in low CO_2_ tension which induces cerebral vasoconstriction [22]. Here, P9 C57BL/6J mice were anesthetized with isoflurane (Butler Schein Animal Health Supply, Reno, NV) (5% for induction, 3% for maintenance) in 2:1 nitrous oxide/oxygen. The body temperature of the pups was maintained at 36 °C using a heated surgical table (Molecular Imaging Products, Bend, OR). Under a surgical microscope (Nikon SMZ-800 Zoom Stereo, Nikon, Melville, NY), a midline neck skin incision was made followed by elevation of the submandibular salivary glands bilaterally. The left carotid sheath was then visualized between the trachea and the left sternocleidomastoid muscle. The left common carotid artery was freed from the carotid sheath by blunt dissection, electrocauterized with a bipolar electrocoagulator (Vetroson v-10 b1-polar electrosurgical unit, Summit Hill Laboratories, Navesink, NJ) and cut. The surgical site was flushed with 0.5% bupivacaine for pain control and closed with a single 6-0 silk suture. Mice were returned to their home cages, which were then placed into a normoxic chamber with a temperature set point of 36.5 ℃ and monitored continuously for a 2-h recovery period. To induce unilateral hypoxic ischemic reperfusion injury, the mice were placed in a hypoxic chamber (BioSpherix Ltd, Redfield, NY) equilibrated with 10% O2 and 90% N2 at 36.5 ℃ for 50 min. After hypoxic exposure, mice were placed in a 36.5 ℃ normoxic chamber for a 2-h recovery period with their dam. Sham-operated mice received anesthesia and exposure of the left common carotid artery without electrocauterization or hypoxia induction as previously described [18].

### Drug administration

**Fig. 1:**
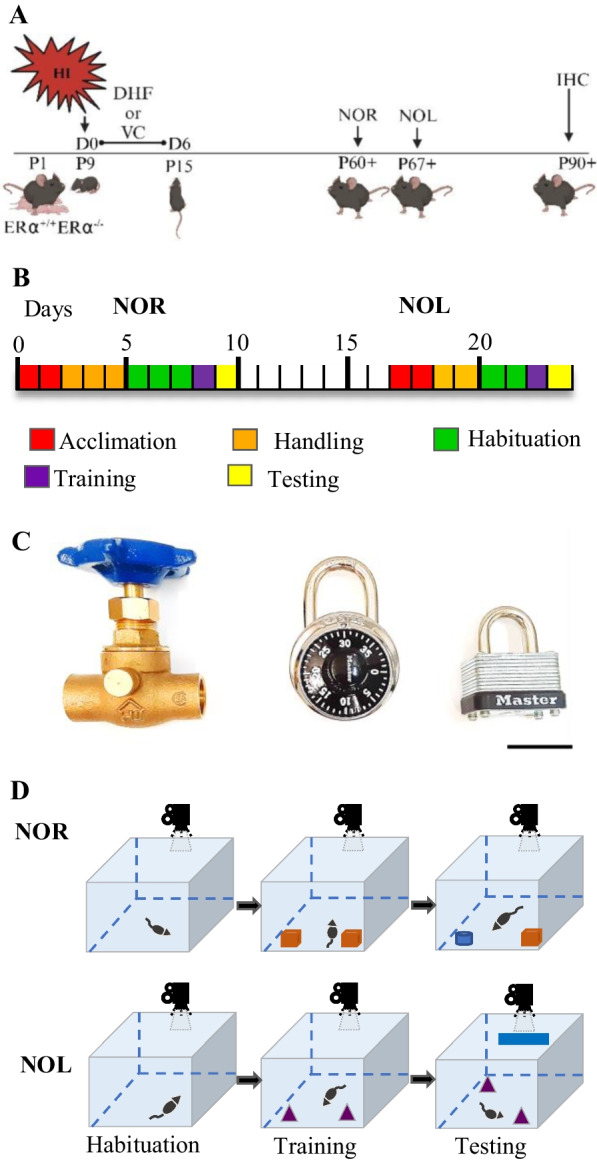
Experimental design.** A** At postnatal day 9 (P9), $\text{ER}\alpha$^+/+^ and $\text{ER}\alpha$^−/−^ male and female mice were exposed to HI mice were either treated with DHF or VC starting 10 min from the HI for 7 days. Mice were assessed with the NOR and NOL tests starting at P60 + , one week apart. Their brains were then perfused fixed at P90 + for IHC staining. **B** Behavioral testing timeline. Prior to behavioral testing, mice were acclimated to the housing and their cages for two days (red). Each test block consisted of acclimation (red), handling (orange), habituation (green), training (purple), and either NOR or NOL (yellow). NOR and NOL tests were spaced one week apart. **C** Representative objects used in behavioral testing were (from left to right): a brass gate valve, a combination lock, and a padlock. Scale bar = 3 cm. **D** Testing apparatus. A white opaque Plexiglas® open field box was used for testing. During habituation, no objects are placed in the open field box. During training two identical objects were placed in the box. In NOR testing, one of the identical objects was replaced with a novel one, whereas, in NOL one of the identical objects was moved to a novel location. During NOL a "clue tape" was placed on the wall of the open field box that served as a spatial orientation cue. All the phases were recorded with an overhead video camera

In studies of DHF administration, male and female littermates were randomly divided into HI-vehicle control (HI + VC) and HI + DHF groups. DHF was dissolved in DMSO at a concentration of 3 mg/mL and frozen as aliquots for up to a month. On the day of use, the DHF was diluted to 0.1 mg/mL with sterile PBS and administered at a final concentration of 5 mg/kg intraperitoneally. The HI + DHF treated mice received the initial dose of DHF at 10 min post-HI. Subsequently, mice were given daily dose of DHF (5 mg/kg) for 6 days for a total of 7 doses. The HI + VC groups and sham + VC received an equal volume of PBS at the same time points for the same duration (Fig. 1A).

### Novel object recognition (NOR) and novel object location (NOL) tests

To minimize possible interfering effects of corticosteroids and other hormones on behavior, we used NOR and NOL tests to assess hippocampus-dependent learning and memory due to their low stress nature that lacks external motivation, reward, and punishment [23]. Mice have an inherent preference for novelty, leading them to interact with novel objects or familiar objects in novel locations. Thus, mice with intact recognition and location memory will spend more time with a novel object or a previously investigated object in a new location. It should be noted that the recognition memory appears to rely on several different brain regions, including the insular cortex [24], perirhinal cortex [25], ventromedial prefrontal cortex [26], and hippocampus [27].

Behavioral testing commenced at P60 + (Fig. 1A) starting with the NOR test and followed approximately one week later by the NOL test (Fig. 1B) [28]. To eliminate potential litter bias due to differential litter susceptibility to HI injury, we included different experimental groups (sham, HI + VC and HI + DHF) and genotype ($\text{ER}\alpha$^+/+^ and $\text{ER}\alpha$^−/−^ mice) from different litters. The behavioral protocols consisted of acclimation, habituation, training and testing phases as shown in Fig. 1B. Raw video footage of the testing phases were analyzed by a blinded experimenter using a software stopwatch (Minute by Minute 2.0.4, Vogue Mechanics Software).

#### Handling

Mice underwent an acclimation period of at least 2 days to acquaint them to their surroundings prior to initiation of NOR and NOL handling. Mice were handled by the experimenter (5 min/day) for the 3 days prior to the beginning of habituation (Fig. 1B). Handling consisted of placing the mouse on the experimenter's non-dominant forearm and allowing the mouse to explore until it relaxed (5 min). The mouse was then returned to its dam. To acclimate mice to objects, a Lego® brick was placed in their home cage during both handling and habituation phases [29, 30].

#### Habituation

All behavioral work was performed in a quiet room under ~ 84 lx lumens of overhead light. Habituation, training, and testing were conducted in an open field box (60 × 60 × 47 cm) constructed of white opaque Plexiglas®. Mice were kept in their cages for 30 min in the testing room followed by habituation. Habituation consisted of allowing the mouse to move freely around the box without an object present for two 10 min sessions, 2 h apart. Habituation was performed for three consecutive days before NOR and 2 consecutive days before NOL. Videos were recorded using a video camera mounted on a tripod that provided a bird’s-eye view of the box. The training phase began 22 h after the habituation phase (Fig. 1B).

#### Training

The duration and number of sessions for training and testing phases in NOR and NOL tests have differed between studies [31]. In many studies, the duration of training and testing sessions is 5 min. However, because HI may alter the normal anxiety-like behaviors both in human neonates [32] and rodents, longer duration of training and testing seemed warranted [33]. For this reason, training and testing times were set as 10 min as previously described [34].

To minimize potential odor cues during testing, three copies of each object were used, and objects were wiped with 70% ethanol between uses. For NOR, one metal padlock and one brass water valve were used as objects (Fig. 1C). For NOL, two identical combination locks were used as objects (Fig. 1C). The identical objects were placed 8 cm from the upper left and upper right corners of the open field box to allow for exploration of all sides of the objects (Fig. 1D)[19]. The mouse was then placed at the mid-point of the wall in-between the objects, with its body parallel to the sidewalls and nose pointing away from the objects, and then released. Actions that constituted exploration included sniffing or touching the objects with nose or forepaws or having the animal stand 2 cm or less away from objects while their nose was directed towards the object. Climbing over or sitting on an object or chewing the object was not considered an explorative behavior unless that action was accompanied with nose-directing behavior toward the object. After 10 min, the mouse was returned to its home cage.

#### Testing

Testing occurred 24 h later following training. After acclimatizing the mice to the testing room and surroundings for 30 min, one of the familiar objects was replaced with a novel object or location for NOR and NOL tests, respectively. Mice were then placed in the open field box and allowed to explore for a total of 10 min (Fig. 1D). Total exploration time during the testing phase and the discrimination ratio (ratio of the time spent with the novel object/location to total time spent with both objects) were reported. Mice that remember the identity and location of the familiar objects will have discrimination ratios higher than 0.5. Mice that had no interest in exploring the objects and did not move were excluded from the analysis.

### Open field (OF) test

For OF test mice were allowed to freely move within the OF box (60 × 60 × 47 cm) constructed of white opaque Plexiglas® for 10 min to examine the exploratory and anxiety-like behavior in rodents [35–37]. Each mouse was placed in the center of the arena and videos were recorded using a video camera mounted on a tripod that provided a bird-eye view of the box. Total distance traveled by mice, percent (%) time spent in the center of the open field, speed of the mice and freezing episodes were reported using ANY-maze program (Stoelting Co.). While all mice underwent open field testing, in a subset of mice the OF test was used as the first session of the habituation phase of the NOR test.

### Immunohistochemistry

Random cohorts of mice in each experimental group that completed OF test were perfused-fixed in situ as described previously [18]. Mice were anesthetized with isoflurane, transcardially perfused with 4% paraformaldehyde, decapitated, and brains were removed. Brains were then post-fixed in 4% paraformaldehyde overnight and cryoprotected in an antifreeze solution (30% sucrose/PBS solution) for storage at -20˚C until they were sliced into 35 µm coronal sections using a frozen sliding microtome (Leica SM2000R, Buffalo Grove, IL). Three slices from each brain (anterior [0.26–0.02 mm bregma; middle [− 1.34–2.06 mm bregma] and posterior [− 2.46–3.16 mm bregma) were rinsed (3 X 10 min) with Tris-buffered saline (TBS) and then blocked in TBS^++^ (0.1% Triton X-100 and 3% goat serum in 0.1 M TBS) for 60 min at 37 ºC as described previously. After blocking, slices were incubated with mouse monoclonal anti-MAP2 (1:500) for 60 min at 37ºC and then overnight at 4 ºC. After rinsing with TBS (3 X 10 min), brain sections were incubated with either goat anti-mouse Alexa Fluor 488-conjugated IgG (1:200) or goat anti-mouse Alexa Fluor 546-conjugated IgG (1:200) for 60 min at 37 ºC. Slices were then washed with TBS (3 X 10 min) and mounted on slides using Vectashield with DAPI. Subsequently, whole brain stitched images of MAP2 staining were acquired. Some slides were imaged with a Leica DMIRE 2 (Leica Inc, Buffalo Grove, IL) inverted epifluorescent microscope using a 5X objective and either a FITC or TRITC filter set. Approximately 72 images were automatically collected per slice and stitched together using the Leica microscope software to produce a whole brain image. In some slides, similar whole brain images were acquired and stitched into a whole brain image using either a Keyence (BZ-X800E, Keyence Corp., Itasca, IL) epifluorescence microscope with a 4 × objective or a Nikon Eclipse Ti2 (Nikon Corp., Melville, NY) epifluorescent with a 4 × objective.

### MAP2 staining and percent area loss measurements

To calculate percent area loss using MAP2 staining, one slice per mouse from anterior, middle and posterior portions of the brain were stained with MAP2 as described above [38]. MAP2-stained individual images were opened in Image J [38]. Using the polygon tool, four regions (hemisphere, hippocampus, cortex, and caudoputamen; see Fig. 4) were traced for both contralateral (CL) and ipsilateral (IL) sides and duplicated. The eight duplicated images were then converted to 8 bits using the Image/Type tool. Then, using the Image/Adjust/Threshold tool, the threshold was adjusted on each image to isolate MAP2 staining in each section. The area of MAP2 staining was then calculated using Analyze/Measure tool in Image J. Hippocampal area measurements were determined in the middle and posterior slices only, caudoputamen area measurements were determined in anterior and middle slices only. The percent change in CL area (CL_a_) versus IL area (IL_a_) for each of the four regions was calculated as (CL_a_ − IL_a_)/CL_a_ × 100).

### Statistical analysis

Behavioral testing outcomes (total exploration time during the testing phase, the time spent with the novel object/location over the first 30 s of accumulated exploration time and the discrimination ratio) and immunohistochemistry outcomes were summarized in terms of means ± standard error (SEM) and stratified by experimental condition (treatment, genotype and sex). We used a three-way analysis of variance (ANOVA) to evaluate differences in behavioral testing and immunohistochemistry outcomes between experimental conditions. Two-way and three-way interaction effects were evaluated. To evaluate treatment-genotype specific differences between males and females, sliced three-way interaction effect contrasts were constructed and analyzed. Residual plots and normal probability plots were examined to verify the model assumptions. The associations between mortality rates, genotype and sex were evaluated using a generalized linear model with a logit link function. All reported P-values are two-sided and *p* < 0.05 was used to define statistical significance. Statistical analyses were conducted using SAS software (SAS Institute, Cary, NC) version 9.4.

## Results

### Restoration of object recognition memories post-HI were $\text{ER}\alpha$-dependent and TrkB-mediated in female mice only

**Fig. 2:**
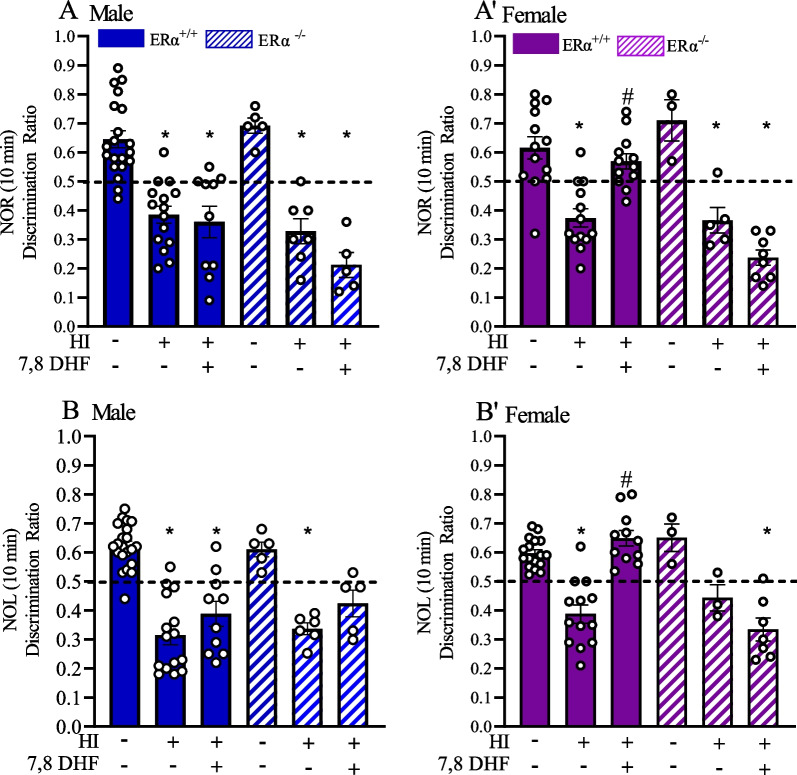
7,8 DHF therapy rescues recognition and location memory only in females in an $\text{ER}\alpha$-dependent way.** A**, **A'** Mice were subjected to NOR testing P60 + post-HI. The discrimination ratio during the first 10 min of NOR testing in males (**A)** and females (**A'**) is shown for $\text{ER}\alpha$^+/+^_sham+VC_, $\text{ER}\alpha$^+/+^_HI+VC_, $\text{ER}\alpha$^+/+^_HI+DHF_, $\text{ER}\alpha$^−/−^_sham+VC_, $\text{ER}\alpha$^−/−^_HI+ VC_, and $\text{ER}\alpha$^−/−^_HI+DHF_ groups. Data are mean ± SEM. *p < 0.05 compared to corresponding sham. #*p* <  0.05 compared to corresponding male $\text{ER}\alpha$^+/+^_HI+DHF_. Significance was analyzed using multivariant ANOVA. **B**, **B'** Mice were subjected to NOL testing P60 + post-HI. The discrimination ratio during the first 10 min of NOR testing in males (**B)** and females (**B')** is shown for $\text{ER}\alpha$^+/+^_sham+VC_, $\text{ER}\alpha$^+/+^_HI+VC_, $\text{ER}\alpha$^+/+^_HI+DHF_, $\text{ER}\alpha$^−/−^_sham+VC_, $\text{ER}\alpha$^−/−^_HI+ VC_, and $\text{ER}\alpha$^−/−^_HI+DHF_ groups. Dotted line at 0.5 shows no preference for the novel object while below the line shows familiar object preference. Data are mean ± SEM. *p<  0.05 compared to corresponding sham. #p< 0.05 compared to corresponding male $\text{ER}\alpha$^+/+^_HI+DHF _Significance was analyzed using multivariant ANOVA.

To determine the role of $\text{ER}\alpha$ in TrkB-mediated long-term recognition memory, we tested $\text{ER}\alpha$^+/+^ and $\text{ER}\alpha$^−/−^ mice at P60 + using NOR test after exposing the mice to HI at P9 (Fig. 1). In the experimental groups tested for NOR, while there was a trend for the treatment by sex by genotype interactions, they were not statistically significant (*F*_(2,104)_ = 2.4, *p* = 0.097). DRs in male $\text{ER}\alpha$^+/+^_HI+VC_ (0.38 ± 0.03) and $\text{ER}\alpha$^+/+^_HI+DHF_ (0.36 ± 0.05) mice were both statistically significantly different than male $\text{ER}\alpha$^+/+^_sham+VC_ (0.64 ± 0.03) mice (*p* < 0.001 and *p* < 0.001, respectively) (Fig. 2A). Thus, DHF therapy did not improve recognition memory to sham levels. In addition, $\text{ER}\alpha$^+/+^_HI+VC_ and $\text{ER}\alpha$^+/+^_HI+DHF_ male mice favored the familiar object more so than the novel object. Contrary to males, although the DRs in female $\text{ER}\alpha$^+/+^_HI+VC_ (0.38 ± 0.03) mice were statistically significantly different than female $\text{ER}\alpha$^+/+^_sham+VC_ (0.62 ± 0.04) mice (*p* < 0.001), the female $\text{ER}\alpha$^+/+^_HI+DHF_ mice DRs (0.57 ± 0.03) did not differ from the $\text{ER}\alpha$^+/+^_sham+VC_ (*p* = 0.34) (Fig. 2A’). Thus the DHF therapy improved the recognition memory to sham levels in the female $\text{ER}\alpha$^+/+^_HI+DHF_ mice only (Fig. 2A’).

The DRs of mice that lack $\text{ER}\alpha$ were then assessed to determine the effect of $\text{ER}\alpha$ in TrkB-mediated sustained neuroprotection post-HI. DRs for male $\text{ER}\alpha$^−/−^_HI+VC_ (0.33 ± 0.03) and $\text{ER}\alpha$^−/−^_HI+DHF_ (0.21 ± 0.04) mice were statistically significant than the male $\text{ER}\alpha$^−/−^_sham+VC_ (0.69 ± 0.02) mice (p < 0.001 and p < 0.001, respectively) (Fig. 2A). Similarly, the DRs for the female $\text{ER}\alpha$^−/−^_HI+VC_ (0.37 ± 0.03) and female $\text{ER}\alpha$^−/−^_HI+ DHF_ (0.24 ± 0.03) mice were statistically significant from the female $\text{ER}\alpha$^−/−^_sham+VC_ (0.71 ± 0.07) mice (*p* = 0.001 and *p* < 0.001, respectively) (Fig. 2A'). Interestingly, DHF therapy did not improve the recognition memory to sham levels in the female $\text{ER}\alpha$^−/−^_HI+DHF_ mice suggesting that this effect tends to be $\text{ER}\alpha$ dependent (0.33±0.02) (Fig. 2A').

### Restoration of object location memory post-HI was $\text{ER}\alpha$-dependent and TrkB-mediated in female mice only

To determine the role of $\text{ER}\alpha$ in TrkB-mediated long-term location memory, we tested $\text{ER}\alpha$^+/+^ and $\text{ER}\alpha$^−/−^ mice at P60 + using NOL test after exposing the mice to HI at P9 (Fig. 1). In contrast to the NOR testing, experimental groups tested for NOL showed statistically significant treatment by sex by genotype interactions (*F*_(2,103)=_11.3, *p* < 0.0001). DRs of male $\text{ER}\alpha$^+/+^_HI+VC_ (0.32 ± 0.04) and $\text{ER}\alpha$^+/+^_HI+DHF_ (0.39 ± 0.03) mice were both statistically significantly different than male $\text{ER}\alpha$^+/+^_sham+VC_ (0.62 ± 0.01) mice (*p* < 0.001 and *p* < 0.001, respectively) (Fig. 2B). Thus, the DHF therapy did not improve the location memory to sham levels and $\text{ER}\alpha$^+/+^_HI+VC_ and $\text{ER}\alpha$^+/+^_HI+DHF_ male mice favored the familiar location more so than the novel location. Contrary to males, although the DRs in female $\text{ER}\alpha$^+/+^_HI+VC_ (0.39 ± 0.03) mice were statistically significantly different than female $\text{ER}\alpha$^+/+^_sham+VC_ (0.60 ± 0.01) mice ((*p* < 0.001), the female $\text{ER}\alpha$^+/+^_HI+DHF_ mice DRs (0.65 ± 0.04) did not differ from the $\text{ER}\alpha$^+/+^_sham+VC_ (*p* = 0.16) (Fig. 2B’). Thus, the DHF therapy recovered the location memory to sham levels in the female $\text{ER}\alpha$^+/+^_HI+DHF_ mice only (Fig. 2B’).

The DRs of mice that lack $\text{ER}\alpha$ were then assessed to determine the effect of $\text{ER}\alpha$ in TrkB-mediated sustained neuroprotection post-HI. DRs for male $\text{ER}\alpha$^−/−^_HI+VC_ (0.34 ± 0.05) and $\text{ER}\alpha$^−/−^_HI+DHF_ (0.42 ± 0.03) mice were statistically significant than the male $\text{ER}\alpha$^−/−^_sham+VC_ (0.61 ± 0.04) mice (*p* < 0.001 and *p* = 0.002, respectively) (Fig. 2B). The DRs of the female $\text{ER}\alpha$^−/−^_HI+VC_ (0.44 ± 0.06) and female $\text{ER}\alpha$^−/−^_HI+ DHF_ (0.33 ± 0.03) were significantly statistically different from the female $\text{ER}\alpha$^−/−^_sham+VC_ (0.65 ± 0.02) mice (*p* = 0.009 and *p* < 0.001, respectively) (Fig. 2B'). Interestingly, the DHF therapy did not improve the location memory to sham levels in the female $\text{ER}\alpha$^−/−^_HI+DHF_ mice indicating the effect is dependent on the presence of $\text{ER}\alpha$ (Fig. 2B').

To determine whether there were group differences in exploring the given object during testing, we analyzed the total exploration times between the groups during NOR and NOL testing (Table 1). While there were no significant differences between the sexes or treatment during NOR testing, in NOL testing male $\text{ER}\alpha$^+/+^_HI+VC_ mice (78 ± 6 s) explored significantly more than female $\text{ER}\alpha$^+/+^_HI+VC_ mice (58 ± 6 s) (*p* = 0.03) with an effect size of 1. In contrast, in mice lacking $\text{ER}\alpha$, there was an effect size as measured by Cohen’s d, of 1.3 between the sexes within the $\text{ER}\alpha$^−/−^_HI+VC_ group, albeit not a significant difference between their exploration times (male 58 ± 5 s vs female 92 ± 25 s, *p* = 0.13) (Table 1).

**Table 1:** Total exploration time (s) accumulated during NOR and NOL testing phases

Total exploration time (s)				
Treatment group	Male	Female	Effect size	P value
NOR				
$\text{ER}\alpha$^+/+^_sham+VC_	81 ± 7	75 ± 8	0.2	0.56
$\text{ER}\alpha$^+/+^_HI+VC_	78 ± 8	79 ± 6	0	0.91
$\text{ER}\alpha$^+/+^_HI+DHF_	91 ± 14	100 ± 10	0.2	0.62
$\text{ER}\alpha$^−/−^_sham+VC_	66 ± 13	60 ± 19	0.2	0.79
$\text{ER}\alpha$^−/−^_HI+VC_	88 ± 16	73 ± 21	0.3	0.63
$\text{ER}\alpha$^−/−^_HI+DHF_	90 ± 10	85 ± 14	0.2	0.78
NOL				
$\text{ER}\alpha$^+/+^_sham+VC_	70 ± 5	67 ± 5	0.2	0.64
$\text{ER}\alpha$^+/+^_HI+VC_	78 ± 6	58 ± 6	1	0.032
$\text{ER}\alpha$^+/+^_HI+DHF_	74 ± 15	80 ± 10	0.2	0.71
$\text{ER}\alpha$^−/−^_sham+VC_	51 ± 9	51 ± 8	0	0.99
$\text{ER}\alpha$^−/−^_HI+VC_	58 ± 5	92 ± 25	1.3	0.13
$\text{ER}\alpha$^−/−^_HI+DHF_	78 ± 13	65 ± 9	0.6	0.42

### Familiarity preference following HI was not associated with anxiety-like behavior

**Fig. 3:**
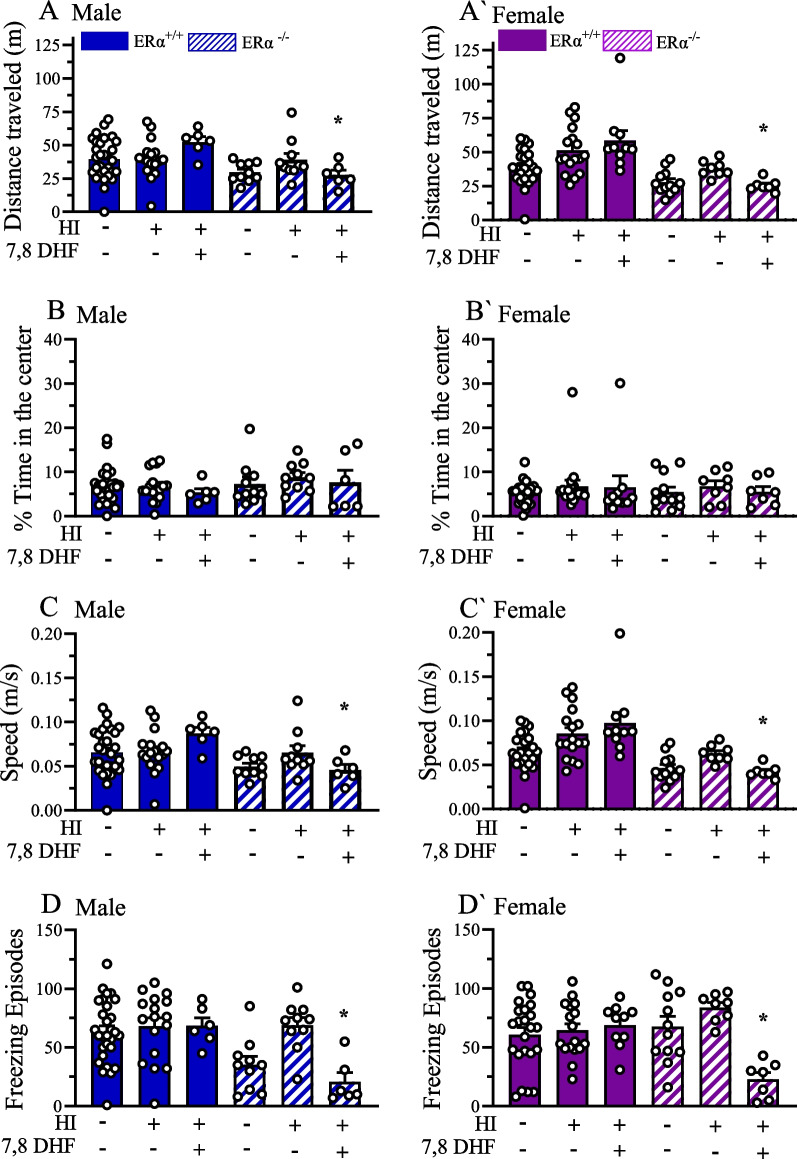
Familiarity preference following HI does not originate from anxiety-like behavior. Mice were subjected to 3 days of habituation prior to NOR/NOL testing. Videos of the 10-min habituation phase on the first day of habituation were analyzed. Distance traveled in male (**A**) and female (**A'**) mice, percent time spent in the center of the open field for male (**B**) female (**B'**), mice and average speed for male (**C**) and female (**C'**), and freezing episodes (**D** and **D'**) of the mice are shown for $\text{ER}\alpha$^+/+^_sham+VC_, $\text{ER}\alpha$^+/+^_HI+VC_, $\text{ER}\alpha$^+/+^_HI+DHF_, $\text{ER}\alpha$^−/−^_sham+VC_, $\text{ER}\alpha$^−/−^_HI+ VC_, and $\text{ER}\alpha$^−/−^_HI+DHF_ groups. Data are mean ± SEM. *p < 0.05 compared to corresponding male or female $\text{ER}\alpha$^+/+^_HI+DHF_. Significance was analyzed using multivariant ANOVA

**Fig. 4:**
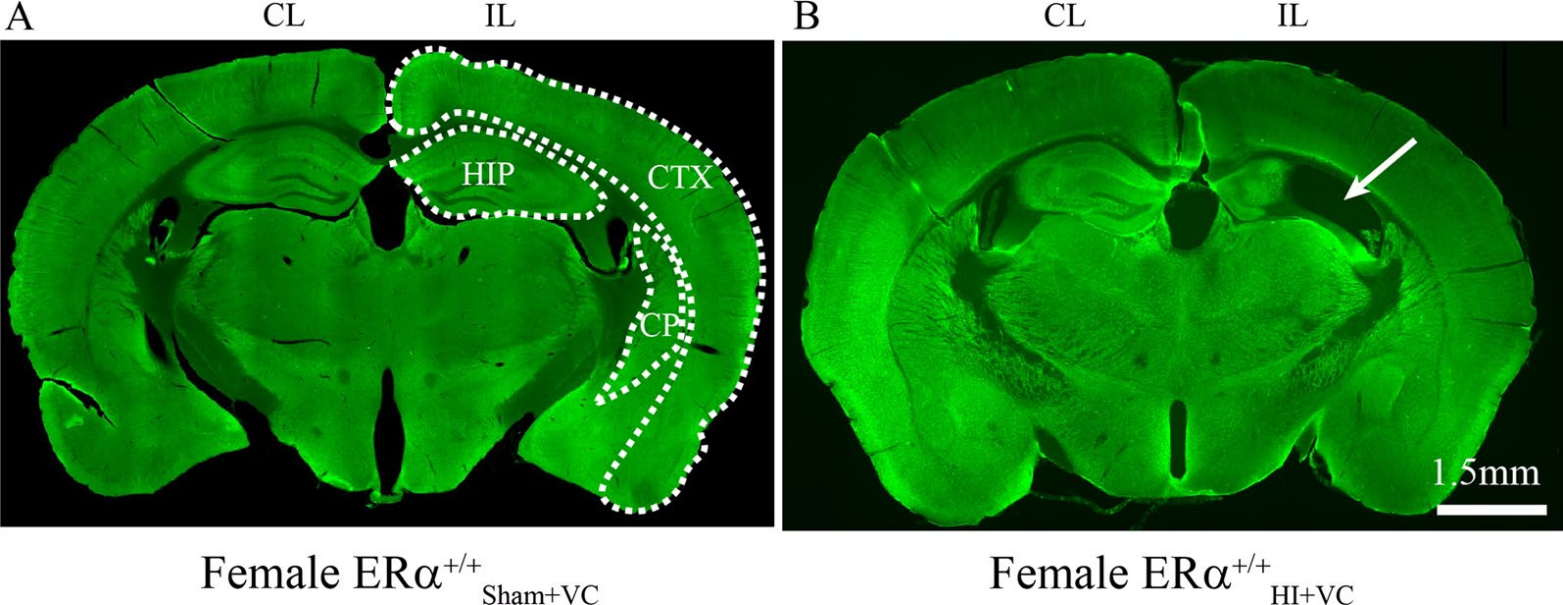
Perinatal HI in mice results in tissue loss at mice at P90 + . Representative coronal images of MAP-2 staining in female $\text{ER}\alpha$^+/+^_sham+VC_ (**A**) and female $\text{ER}\alpha$^+/+^_HI+VC_ (**B**) mice at P90 + . Regions of interest are presented as dotted tracings. Contralateral CL, ipsilateral (IL). *HIP* hippocampus, *CTX* cortex, *CP* caudoputamen. Arrow = hippocampal tissue loss. Scale bar = 1.5 mm

In NOR and NOL tests, male and female $\text{ER}\alpha$^+/+^_HI+VC_, and male $\text{ER}\alpha$^+/+^_HI+DHF_ mice demonstrated preference for either the familiar object or location. We asked the question of whether this familiarity preference is due to HI-induced anxiety or not. Thus, we performed OF testing to assess the anxiety-like behavior in $\text{ER}\alpha$^+/+^ and $\text{ER}\alpha$^−/−^ mice at P60 + after exposing the mice to HI at P9 (Fig. 1). We analyzed; [1] total distance traveled (Fig. 3A, A’); [2] % time spent in the center of the open field (Fig. 3B, B’); (3) speed of the mice (Fig. 3C, C’), and (4) freezing episodes (Fig. 3D, D’). Our results showed that, male $\text{ER}\alpha$^+/+^_HI+DHF_ mice explored statistically significantly greater distances (*p* = 0.034) and moved faster (*p* = 0.033) but froze more (*p* = 0.01) than male $\text{ER}\alpha$^−/−^_HI+ DHF_ mice (Fig. 3A–D). Similarly, female $\text{ER}\alpha$^+/+^_HI+DHF_ mice explored statistically significantly greater distances (*p* < 0.001) and moved faster (*p* = 0.001) but froze more (*p* = 0.003) than $\text{ER}\alpha$^−/−^_HI+ DHF_ mice (Fig. 3A'–D'). These results suggest that DHF therapy in $\text{ER}\alpha$^−/−^ mice increased anxiety-like behavior when assessed by distance traveled and speed in open field test but decreased anxiety when assessed by freezing. No sex by experimental group effect was detected in the OF test.

### TrkB agonist therapy did not reduce HI-induced tissue loss

**Fig. 5:**
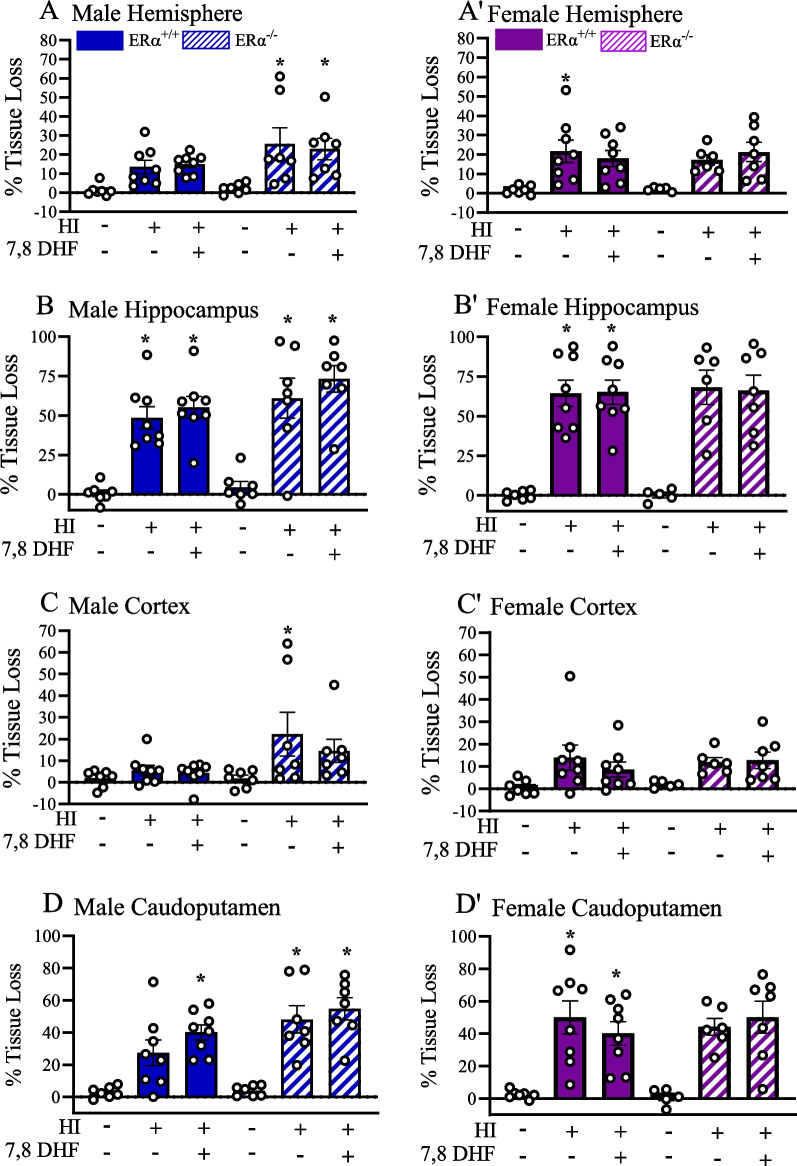
Percent hemispheric, hippocampal, cortical and caudoputamen tissue loss in mice at P90 + following perinatal HI. Percent hemispheric tissue losses in male (**A**) and female (**A'**) hemispheric, male (**B**) and female (**B'**), hippocampus, male (**C**) and female (**C'**) cortex and male (**D)** and female (**D'**) caudoputamen (**D**,** D'**) determined by MAP2 staining. Data are mean ± SEM. *p < 0.05 compared to corresponding sham. Significance was analyzed using multivariant ANOVA

After completing behavioral tests around postnatal day 90 + , we fixed brains for MAP2 staining, and percent tissue loss was calculated in both $\text{ER}\alpha$^+/+^ and $\text{ER}\alpha$^−/−^ mice. We aimed to identify a region or regions that were protected and may have contributed to the recovery of the recognition and location memories following HI in female $\text{ER}\alpha$^+/+^ mice but not in $\text{ER}\alpha$^−/−^ mice. The region of interests (ROI) included hemisphere (as a measure of global brain injury), hippocampus, cortex and caudoputamen (Fig. 4). There was no sex effect on post-HI, hemispheric tissue loss (*F*_(5,69)_ = 0.79, *p* = 0.56). In addition, there was no statistically significant difference between the $\text{ER}\alpha$^+/+^_sham+VC_ and $\text{ER}\alpha$^+/+^_HI-VC_ male and female mice. There were also no statistically significant sex differences found between the male (13.4 ± 3.5%) $\text{ER}\alpha$^+/+^_HI-VC_ and female (21.7 ± 5.7%) $\text{ER}\alpha$^+/+^_HI-VC_ mice (Fig. 5A, A’). The hippocampus was found to be the most vulnerable area post-HI among the 3 regions. Compared to their shams, percent hippocampal tissue loss was significantly higher in male $\text{ER}\alpha$^+/+^_HI-VC_ (48.6 ± 7.0%, *p* = 0.006) and in female $\text{ER}\alpha$^+/+^_HI-VC_ (64.5 ± 8.3%, *p* = 0.021) mice (Fig. 5B, B’). However, in cortex no statistically significant percent tissue loss was detected in male $\text{ER}\alpha$^+/+^_HI-VC_ (5.5 ± 2.4%, *p* = 0.99) and in female $\text{ER}\alpha$^+/+^_HI-VC_ (13.9 ± 6.1%, *p* = 0.13) mice (Fig. 5C, C’). Compared to their shams, percent caudoputamen tissue loss was significantly higher in female $\text{ER}\alpha$^+/+^_HI-VC_ (50.0 ± 10.1%, *p* = 0.002) mice, but did not reach statistical significance in the male $\text{ER}\alpha$^+/+^_HI-VC_ (27.3 ± 8.0%, *p* = 0.062) mice (Fig. 5D, D’). No statistically significant sex effects were detected in percent hemispheric, hippocampal, cortical and caudoputamen tissue loss between the male and female $\text{ER}\alpha$^+/+^_HI-VC_ mice. In addition, DHF therapy had no effect on the percent hemispheric, hippocampal, cortical and caudoputamen tissue loss in the male and female $\text{ER}\alpha$^+/+^ mice.

In $\text{ER}\alpha$^−/−^ mice, HI resulted in a significant loss of hippocampal tissue in male (61.1 ± 12.7%, *p* < 0.001), whereas the hippocampal loss in female mice did not reach significance (68.2 ± 10.8%, *p* = 0.08) compared to their shams. As with the $\text{ER}\alpha$^+/+^ mice, treatment with DHF did not decrease the percent tissue loss in the hippocampus in either male or female $\text{ER}\alpha$^−/−^ mice. In a similar matter, there was a significant loss of male caudoputamen (48.2 ± 9.0%, *p* < 0.001) in $\text{ER}\alpha$^−/−^ mice, but not in female mice (44.2 ± 5.2%, *p* = 0.52). DHF treatment did not significantly affect any regions in $\text{ER}\alpha$^−/−^ mice.

### Complete elimination of $\text{ER}\alpha$ predisposes neonatal mice to greater mortality

In order to determine the effect of HI and the presence or absence of $\text{ER}\alpha$ on mortality, we determined the mortality rates of $\text{ER}\alpha$^+/+^ and $\text{ER}\alpha$^−/−^ mice post-HI (Table 2). Compared to $\text{ER}\alpha$^+/+^ male mice, $\text{ER}\alpha$^−/−^ male mice had a significantly higher mortality rate during surgery (*p* = 0.001), hypoxia (*p* = 0.03) and post-HI (*p* = 0.03). Similarly, compared to $\text{ER}\alpha$^+/+^ female mice, $\text{ER}\alpha$^−/−^ female mice had significantly higher mortality rate during surgery (*p* = 0.02) and post-HI (*p* = 0.01). Overall, $\text{ER}\alpha$^−/−^ mice had a statistically significant higher mortality rate than $\text{ER}\alpha$^+/+^ mice (*p* < 0.0001).

**Table 2:** Comparison of the mortality rates of $\text{ER}\alpha$^+/+^ and $\text{ER}\alpha$^−/−^mice during surgery, hypoxia, and post-HI

Mortality	$\text{ER}\alpha$^+/+^ (*n*)		$\text{ER}\alpha$^−/−^ (*n*)	
	Male (68)	Female (52)	Male (32)	Female (28)
During surgery	0	2	6	6
During hypoxia	0	0	3^*^	1
Post-HI	2	0	5^*^	4^*^
Total	2	2	14^*^	11^*^

^*^p < 0.05 compared to $\text{ER}\alpha$^+/+^ using a generalized linear model with a logit link function

## Discussion

We have previously reported that HI induced in mice during the neonatal period increases hippocampal TrkB phosphorylation which was further augmented only in females by the TrkB agonist/modulator, DHF, resulting in early neuroprotection in an $\text{ER}\alpha$-dependent manner [13]. However, whether this sex-specific $\text{ER}\alpha$-dependent TrkB-mediated neuroprotection is sustained into early adulthood is unknown [18]. In this study, we assessed the role of $\text{ER}\alpha$-dependent TrkB-mediated neuroprotection in recognition memory, location memory and anxiety-related behavior in young adult mice after neonatal HI. Our hypothesis was that TrkB-mediated long-term neuroprotection post-HI is female-specific and $\text{ER}\alpha$-dependent. Our hypothesis is supported by our findings which demonstrate that treatment with a TrkB agonist following neonatal HI in females, but not in males, improves learning and memory and sustains into early adulthood. Importantly, these patterns seen in recognition and location memories tended to be or were absent in female mice lacking $\text{ER}\alpha$, respectively.

### Therapeutic hypothermia and neurodevelopmental outcomes following neonatal HI in humans

Therapeutic hypothermia is now the standard of care in neonatal intensive care units. If initiated within 6 h of life, the relative risk of mortality drops by 26% [39]. However, the finding of normal neurodevelopment post-neonatal HI does not preclude cognitive and behavioral difficulties in late childhood and adolescence, because cognitive functions are not fully developed at this early age. Indeed, studies show that children who survive HI continue to suffer from cognitive and developmental delays, learning difficulties, and behavioral disabilities [40, 41]. In addition, long-term follow-up studies of therapeutic hypothermia trials failed to show significant improvement in functional activity and IQ scores [42, 43]. In addition, therapeutic hypothermia trials have not examined how sex may influence long-term outcomes of HI in neonates. On the other hand, studies have shown that the long-term neuropathology in pediatric traumatic brain injury and in preterm children is more severe in males than females [42, 44–51]. In addition, animal studies suggest that neurodevelopmental disorders are more common in male neonates compared to females following HI [52–54]. Thus, there is a pressing need to investigate neonatal HI in both sexes to elucidate mechanisms that could lead to sexually specific adjunctive targeted therapies which improve the neurodevelopmental outcomes after HI.

### DHF therapy recovers recognition and location memories in the presence of $\text{ER}\alpha$ only in female mice

In our study, we tested the neuroprotective effect of DHF on recognition and location memories in the presence and absence of $\text{ER}\alpha$ following HI in both males and females. Our findings show that although sham mice had robust recognition and location memories, HI induced a significant decrease in discrimination ratios in both sexes regardless of the presence or absence of $\text{ER}\alpha$. This suggests a disruption of the mice’s innate behavior to investigate novel experiences in their environment. DHF therapy administered after HI for 7 days restored the recognition and location memories in female mice. Notably, DHF effect in location memory was statistically dependent on the presence of $\text{ER}\alpha$. This suggests that $\text{ER}\alpha$ is a necessary component of TrkB-mediated neuroprotection after brain injury that is specific to females. However, it should be noted that the small sample size in some of the experimental groups is a limitation.

Sex differences in learning and memory tests have been reported in animal models of HI, with males suffering more detrimental consequences of HI than females. Using a model of the neonatal HI in the rat, Waddell et al. explored neurogenesis in the hippocampi of male and female neonates (P10) and the ability of estradiol treatment for 2 days post-HI to restore memory at adolescence. P30 post-HI males showed impaired recognition memory in NOR testing using either a short-term (1 h) or long-term (24 h) memory retention paradigm. On the other hand, female recognition memory was not affected by HI. Interestingly, estradiol treatment in males restored recognition memory in males. They also showed that at P30 post-HI there was a decrease in IL hippocampal volume in both sexes which was about 25%. Estradiol treatment had no effect on hippocampal volumes post-HI in either sex [53]. On the other hand, in our model of neonatal HI we tested the mice at P60 + , which corresponds to young adulthood instead of adolescence (P30). We found that at P60, using a long-term retention (24 h) paradigm, both recognition and location memories were impaired in both males and females. This more severe disruption of learning and memory may be related to the severity of the hippocampal injury in our model (50 -75%, Fig. 5). Supporting this hypothesis, after inducing HI in P10–11 rat pups, Patel et al. assessed the effect of therapeutic hypothermia on recognition memory at P56 using a short-term retention (6 min) paradigm. Their results showed that impaired NOR occurred only in rats with severe brain damage (25–100% infarct) with no evidence of protection with therapeutic hypothermia [55]. Thus, sex differences in recognition memory post-HI may depend on the timing of the testing, the retention paradigm used, and the severity of hippocampal injury.

As opposed to NOR, there are very few animal studies that investigate NOL memory post-HI. Diaz et al. exposed mice to HI (45 min, 8% O_2_) at P10 with and without post-HI hypothermia therapy and then tested the mice at P23 using a short-term (30 min) retention paradigm NOL memory test. Interestingly, the only significant NOL deficit post-HI at P23 was in hypothermia treated male mice [56]. In another study, Gilchrist et al. used a model of intrauterine growth restriction (IUGR) at embryonic day 18 (term ~ 22) to produce chronic hypoxia in rat pups. At P36-38 NOL memory was tested in the pups with a short-term retention (5 min) paradigm. While they reported increased hippocampal neurogenesis in the IUGR mice, IUGR did not affect NOL in these mice [57]. Neither of these studies produced severe hippocampal injury. In our study, both sexes exhibited impaired memory at P60 + using a long-term (24 h) retention paradigm of NOL.

### Role of $\text{ER}\alpha$ in TrkB-mediated neuroprotection following HI

In adult rodents, chronic estradiol therapy in ovariectomized female rats after transient global ischemia reduced ischemia-induced neuronal cell loss and improved recognition memory but not location memory [58]. Thus, following brain injury there might be a role for $\text{ER}\alpha$ in adolescent females that promotes recovery. Studies have demonstrated a strong connection between $\text{ER}\alpha$ and TrkB signaling in several animal models ranging from ischemic–reperfusion injury to schizophrenia, all highlighting the important role of this relationship in cognitive deficits and disability susceptibility [59, 60]. $\text{ER}\alpha$ is critical for regulation of the NMDAR-regulated kinases Src, extracellular signal-regulated protein kinase 1/2 (Erk1/2), and TrkB, each of which play critical roles in consolidating newly induced long-term potentiation (LTP) [61]. In adult animal stroke models, $\text{ER}\alpha$ and TrkB signaling has a protective relationship, specifically when $\text{ER}\alpha$ is activated via estradiol [62]. In addition, Gross et al. showed that dorsal hippocampal infusion of the TrkB antagonist ANA-12 prevents 17β-estradiol-induced memory consolidation in the NOR and NOL tasks in ovariectomized adult female mice [63]. Hippocampal memory formation is complex but involves modulation in adults by 17β-estradiol through activation of the classical estrogen receptors $\text{ER}\alpha$ and ERβ [23] as well as the G-protein-coupled estrogen receptor through a 17β-estradiol-independent mechanism [23, 28]. It has also been shown that in adult rat hippocampal acute slices, LTP depends on membrane $\text{ER}\alpha$ in females but not in males, and there is an $\text{ER}\alpha$-dependent increase in postsynaptic TrkB only in females [61]. In future studies, we plan to determine the relative role of membrane and nuclear $\text{ER}\alpha$ in the $\text{ER}\alpha$-dependent TrkB-mediated neuroprotection.

### Mice prefer to explore the familiar object post-HI

In NOR and NOL testing, both male and female $\text{ER}\alpha$^+/+^_sham+VC_, $\text{ER}\alpha$^−/−^_sham+VC_, and female $\text{ER}\alpha$^+/+^_HI+DHF_ mice preferred exploring the novel objects and locations. However, male and female $\text{ER}\alpha$^+/+^_HI_ and $\text{ER}\alpha$^−/−^_HI_ mice, as well as male $\text{ER}\alpha$^+/+^_HI+DHF_ mice demonstrated a significant preference for the familiar objects and locations. These data are consistent with several studies that have also reported a familiarity preference in adult rodents post-HI using short (5 min–1 h) retention paradigms [53, 64–66]. Others have found that the hippocampus plays a significant role in object recognition and that following temporary or permanent lesion of the hippocampus disrupts object recognition when the retention paradigm used is longer than 10 min [67]. While there is a remarkable variability in NOR methods used across studies which can complicate comparisons, the issue of familiarity preference among HI-exposed rodents remains to be fully investigated.

We also examined the relation of anxiety-like behavior and the familiarity preference among mice in the OF testing. We did not see any differences between sham or HI mice either in with or without $\text{ER}\alpha$ in time spent their distance traveled, time in center, speed, or freezing episodes. This would indicate that anxiety, as assessed with these tests, was not a factor in familiarity preference in these animals. An unexpected finding was that DHF treatment in $\text{ER}\alpha$^−/−^ mice, regardless of sex, caused an increase in anxiety, as assessed with distance traveled and speed, but paradoxically a decrease in anxiety, as assessed by freezing episodes. This result needs to be confirmed using additional anxiety tests such as the elevated plus maze or light/dark box tests [36]. Ming-Yan et al. subjected rats to either mild or severe unilateral HI and then performed the EPM test at P14, P21 and P28. Rats with mild and severe HIE at all ages demonstrated decreased anxiety-like behavior compared to the control groups (Ming-Yan et al., 2012). Interestingly, Muntsant et al. showed that in the corner test, HI mice showed a decreased number of corner visits and their latency period of first rearing measurements was higher. Additionally, they reported that HI males were found to be more neophobic as compared to HI females [68]. Arteaga et al. reported that rats subjected to neonatal HI at P90 exhibited increased head dipping behavior suggesting increased anxiety and neophobia behavior in these animals [64]. On the other hand, Duran-Carabali et al. used central crossing as an index of anxiety-like behavior and reported increased impulsivity in male but not female rats at P60 + post-HI [69]. Interestingly, a disruption of aversive memory has been reported using an inhibitory avoidance test in adult rats that had undergone neonatal HI [66, 70]. Thus, understanding the possible causes of familiarity preference in our model will require further investigation into associated anxiety, aversive memory or neophobic behavioral outcomes that can accompany HI.

### $\text{ER}\alpha$-dependent TrkB-mediated long-term neuroprotection in females does not depend on the neuropathology post-HI

In rodent HI models, there is a correlation between the age of the animal post-HI and the severity of tissue loss and functional deficits [71]. In adult rodents, the tissue loss IL to the HI injury is most severe and involves the cortex, caudoputamen and especially the hippocampus resulting in the most severe behavioral deficits [72]. Even so, Smith et al. reported that at P88 + following neonatal HI in rats, there was a significant reduction in the volume of the IL cortex and hippocampus, and a significant increase in the volume of the IL ventricle with no sex difference. Still, they observed males had significant worse behavioral outcomes than female. They suggest that female protection may be the result of sex-specific plasticity or compensation rather than a reduction in gross neuropathology [52]. In our study, that percent tissue loss depended neither on sex nor the presence or absence of $\text{ER}\alpha$ expression. In addition, DHF treatment did not affect the percent tissue loss in any brain regions that play a role in recognition and location memories. Further study is needed to elucidate the effect of therapies on behavioral outcomes and the relationship to the extent of damage to specific brain regions.

### Lack of $\text{ER}\alpha$ increases mortality during and following HI

It should be noted that in our study $\text{ER}\alpha$^−/−^ mice (42%) had significantly higher mortality than $\text{ER}\alpha$^+/+^ mice (3%). A lack of $\text{ER}\alpha$ predisposed mice to die during anesthesia as well as post-HI (Table 2). The higher mortality may be due to the severe hypoxia–ischemia–reperfusion injury to the brain as well as hypoxic injury to other organs that express $\text{ER}\alpha$. To our knowledge, this is the first study to utilize $\text{ER}\alpha$^−/−^ mice during the neonatal period. An unexpected result was the finding that $\text{ER}\alpha$^−/−^ mice are more vulnerable and have a higher overall mortality in both sexes compared to $\text{ER}\alpha$^+/+^ mice post-HI. The $\text{ER}\alpha$ knockout phenotype is primarily characterized by infertility in female and male mice [73]. Other investigators have used $\text{ER}\alpha$^−/−^ mice in studies of adult rodent stroke (focal ischemia), however did not report mortality in their studies although gross brain injury was comparable between knockout and wildtype mice [74, 75]. However, the $\text{ER}\alpha$^−/−^ mice in our study had similar degree of gross tissue loss compared to the wildtype animals and they displayed similar total exploration times, indicating no loss in the ability to investigate during the object and location memory tests (Table 1). Thus, the $\text{ER}\alpha$^−/−^ phenotype would not have a direct effect on the knockout animals DRs.

### Perspectives and significance

Neonatal HI leads to severe life-long morbidities in thousands of neonates born in the US each year [76]. Clinical studies indicate that female neonatal brains are more resistant to the effects of neonatal HI and showing better long-term cognitive outcomes as compared to males [44] suggesting that there might be sex-specific mechanisms that afford females greater neuroprotection. There is a gap in the current literature regarding sex-specific molecular and cellular mechanisms that mediate female-biased neuroprotection in neonates. Our study was designed to examine the hypothesis that long-term cognitive outcomes of neonatal HI may differ between males and females, and that the neuroprotective effects of TrkB agonist therapy on these long-term outcomes may differ by sex and be $\text{ER}\alpha$-dependent. Building on our past and present findings that implicate $\text{ER}\alpha$ in the neurotrophin receptor-mediated neuroprotection in females, we will investigate further the molecular mechanism to understand origins and cellular basis of sex differences in neuroprotection following neonatal HI, and in particular the potential mechanisms by which TrkB agonists may provide a new avenue for therapeutic intervention [18]. We hope that our studies will provide the foundational insights necessary for future development of novel sex-specific therapeutic targets for neonates suffering from HI.

## Conclusion

Our results confirm the validity of: (1) HI impairs long-term recognition and location memories post-HI in both males and females; (2) DHF therapy recovers long-term recognition and location memories only in female mice. Improvement in recognition memories tended to be and location memories are $\text{ER}\alpha$-dependent; (3) lack of $\text{ER}\alpha$ makes male and female neonatal mice more susceptible to HI and results in increased mortality; (4) DHF therapy does not recover the gross pathological injury seen by IHC staining but improves the long-term neuroprotection most probably by restoring the normal neural circuitry in females by alterations at the molecular level downstream of the TrkB-$\text{ER}\alpha$ pathway.

## Data Availability

The data that support the findings of this study are available from the corresponding author upon reasonable request.
